# Birth and Breeding

**Published:** 1882-04

**Authors:** F. M. Holland


### POPULAR SCIENCE DEPARTMENT.

### BIRTH AND BREEDING.

F. M. HOLLAND, in *The Index*.

Several years of special study of the parentage, education, and habits of men and women noted for genius, virtue, or vice, have led me to results which I present as approximations to the truth.

These I gained partly by investigating the lives of nearly seven hundred men and women, most of whom I found mentioned in order in the biographical section of the subject-catalogue of the Harvard College Library, and almost all of whom were born after 1700, and partly from collecting the facts given in Dugdale's "Jukes," Galton's "Hereditary Genius," the "Newgate Calendar," Duyckinck's "Cyclopædia," Brown's "History of the American Stage," Mrs. Hale's "Woman's Record," Regli's "Dizionario Biografico dei Poeti et Artisti," Feuerbach's "Merkwürdige Criminal-Rechtsfälle," North's "History of Augusta, Me.," and a number of smaller works.

I. The point I examined most carefully was the comparative influence of fathers and mothers. I found nearly fifteen hundred cases in which peculiarities of character or ability seemed to be inherited from one parent rather than the other. Among these there were eight hundred and thirty-two instances of the son's genius coming from the father rather than the mother, and only sixty-five of its coming from the mother rather than the father—a difference of about thirteen to one. This disproportion is partly due to omissions of cases of female influence, but I do not think that it is wholly caused thus; for all the collections of biographies give similar results, even in those books in which women receive most attention. This is particularly true of Regli's "Dizionario Biografico," which mentions forty-three cases in which genius seems to be transmitted mainly through the fathers, and but one of apparent derivation from the mother. Brown, too, in his "History of the Stage," gives the parentage of three times as many actresses as actors, but only one instance of mainly maternal influence to fourteen where it seemed principally paternal. And Mrs. Hale, who is so anxious to have women receive all due honor that she gives the same author two separate articles, in which her name and the titles of her books are spelled with slight variations, records but six cases of gifted mothers who were the sole transmitters of ability to their sons. Among other great men who had distinguished fathers and obscure mothers may be mentioned Charlemagne, Dante, Raphael, Galileo, Milton, Mozart, Pitt, and John Stuart Mill. Examining cases in which the children and both parents are known to me personally, I found fourteen boys who are more like their fathers than their mothers mentally, and ten instances of the reverse. Among men remarkable for goodness, seventy-four appeared to be influenced mainly by their fathers and thirty-two by their mothers, the disproportion exceeding two to one. Forty of these men were ministers, giving thirty-two cases of paternal and eight of maternal influence, four to one. I also found eighteen instances in which it was the father and son who were notoriously wicked, and fourteen in which it was mother and son. In all there are nine hundred and thirty-eight cases of the sons being influenced in either character or ability by the father rather than the mother, and one hundred and twenty-one in which the influence is mainly maternal. If more was known about the mothers, this disproportion would not appear so great; still, it would, I think, be great enough to show that the gifted and saintly mother theory rests mainly on courtesy.

The women seemed, in three hundred and forty cases, to have inherited ability mainly from the father, and in fifty-six from the mother, a ratio of six to one, though half of the instances were from books written in praise of female genius. Moreover, I found that, of thirty-one girls whose parents were personally known to me, nineteen took after the father rather than the mother, while but twelve showed the preponderance of maternal influence. The books also give forty cases in which moral influence seemed to come from the father, and thirty-five in which it was rather from the mother. Of the father's superior influence, either mental or moral, over the daughter, there are thus three hundred and ninety-nine cases, and of the mother's one hundred and three.

The whole number of instances of preponderating paternal influence over either the mind or the morals of the son or daughter is thus thirteen hundred and thirty-seven, and of maternal two hundred and twenty-four. The latter is probably incompletely recorded, so that in reality the father's influence is not on the average six times as strong as the mother's. Still, we can easily believe it to be somewhat greater, when we remember how sadly women have been repressed through denial of thorough education, exclusion from learned professions and public office, social pressure against their thinking, and speaking even as freely as men have done, and other forms of oppression. This restraint has been much greater intellectually than morally ; and hence the greater power of the mother to influence the child's morals than its mind, as indicated by my statistics. The fact of such repression, as well as of the superior authority given to the father by law and public opinion, together with the comparative failure—mentioned by Galton and confirmed by my own investigations—of women of genius to marry, justifies the belief that the mother's influence really is, on the average, inferior to the father's, especially over the boys.

II. No account has been taken in the previous section of cases of inheritance of genius from both parents ; for instance, Alexander, Bacon, Goethe, and Napoleon Bonaparte. Sons of clergymen and church members are peculiarly likely to enter the ministry. There are also particularly strong tendencies to inherit idiocy, pauperism, and crime, especially licentiousness and intemperance. In examining the pedigrees of nearly one thousand paupers and criminals in Dugdale's "Jukes," I found that when both parents were vicious four fifths of the children were known to be like them, and scarcely any appear to be much better. One half of the women were licentious. And Mr. Dugdale says that "hereditary pauperism seemed to be even more firmly fixed than hereditary crime." Dr. Thomson, for many years resident surgeon in a Scottish prison, holds that, "in by far the greatest proportion of offences, crime is hereditary," and therefore "it is intractable in the highest degree." (*Journal of Mental Science*, January, 1870, vol. xv., pp. 487–498.) It is of course difficult to distinguish the effects of education from those of parental influence after birth. The most instructive cases are those of children who were removed early from vicious parents and brought up in virtuous and enlightened families. Such children almost invariably turn out better than their parents. At first, they usually show no particularly bad traits ; but, as growth matures, the parental character frequently shows itself, though in a much mitigated form.

The tendency of the child to resemble the parents does not, however, seem sufficiently constant to be called a law. Less than one hundred and forty of the thousand men and women of ability mentioned in Galton's *Inherited Genius* had gifted parents, and less than four hundred had any relatives with much mental power ; so that about two thirds showed no signs of inheritance of intellect. I found in comparing two hundred and nineteen men and women of high ability with two hundred and ninety-two who did not rise above mediocrity, that five times as large a part of the former as of the latter had both fathers and mothers with intellectual gifts and tastes; but I found also that at least forty per cent of these able people were not of remarkable parentage. Among instances of genius which does not appear to have been inherited are Julius Cæsar, Shakespeare, Peter the Great, Frederick the Great, Maria Theresa, George Sand, Jackson, Cavour, Garibaldi, and Lincoln. Brothers and sisters, even twins, are often found to differ so widely as to show great irregularity of parental influence.

III. Among wholly unexpected discoveries was that the larger the number of brothers and sisters, the greater seems to be the probability that some will become highly virtuous. Dividing four hundred and seven of the people whom I studied most carefully into four groups, according to differences in ability as well as in character, I found that the virtuous men and women had, on the average, fifty per cent more brothers and sisters than the vicious of corresponding ability. North's "History of Augusta, Me.," gives all the children in three hundred and twelve families, twenty of which contained a son noted for moral worth, and these twenty families averaged almost exactly eight brothers and sisters, the number in more than one half exceeding nine; while the other two hundred and ninety-two averaged less than six, the number in less than one fifth exceeding nine. Among the people of low ability and character, whom Dugdale calls the Jukes, I found that the comparatively virtuous families averaged six and two thirds children, while the more vicious averaged five and two thirds. I presume that the intercourse of brothers and sisters has a tendency to develop sympathy, and thus promote the growth of conscience.

IV. Similar results might be expected from the meeting together of children in our schools; but the moral value of these institutions has been so decried by Herbert Spencer in "Social Statics," and also by Richard Grant White in the *North American Review* for December, 1880, that the question needs careful study.

Spencer relies mainly on Joseph Fletcher, an eminent British specialist, who more than thirty years ago contributed to the tenth, eleventh, and twelfth volume of the *Journal of the London Statistical Society* three lengthy papers, the last of which is illustrated by thirty elaborate tables and twelve shaded maps. In these papers he divides England and Wales into industrial districts and groups of counties, and shows that the best educated of these—that is, those in which ability to sign the marriage register was least rare—were most free from paupers, commitments for crime, and illegitimate births. Again and again, he speaks of "the generally good influence exerted by the schools," and declares that "the excess of the more heinous and brutal" offences is "always on the side of the greater ignorance." Herbert Spencer takes no notice of frequent statements like these, and simply quotes from Fletcher's conclusion a few introductory sentences, admitting that in some cases, when we take all the commitments of crime together indiscriminately, we can find "a superficial evidence against instruction." What Spencer quotes, however, was simply designed to introduce what he wholly omits; namely, that Fletcher, by separating these commitments into three classes, according to enormity of crime, and placing the more serious offences against the person with the malicious offences against property, especially those accompanied by violence, is able to prove that the worst crimes are most frequent in the most ignorant districts, so that we have "a testimony in favor of the educational influences generally associated with instruction far more powerful than any that has yet been supplied," and "the conclusion is therefore irresistible that education is essential to the security of modern society." Thus Fletcher brings up a long array of arguments in behalf of the moral value of schools, and admits incidentally that some of the statistics appear to disparage this value, but shows that even these, when thoroughly examined, sustain the main conclusion; namely, that public school education promotes moral progress. Herbert Spencer, probably misled by some assistant or reviewer, quotes the admission without its context, and on the strength of it refers to Fletcher as a witness against the truth which his laborious investigations have fully proved. That Herbert Spencer had not himself read Fletcher's papers is all the more likely from the curious circumstance that they furnish good reasons for setting aside almost all the other testimony relied upon in "Social Statics." I do not wish to say too much about a book published thirty years ago, but so great has been and will be its authority, that it is high time for this mistake to be pointed out.

The adverse influence of "Social Statics" is noticed in the recent pamphlet on "Education and Crime," published by the Department of the Interior and written by J. P. Wickersham, who completely refutes the charge that the higher grades of education favor crime, and also gives many encouraging facts: such as that one sixth of the crime in Pennsylvania is committed by the illiterate one thirtieth part of the population; that in

New York City " the chances for crime among those who cannot read and write are nine times as great as among the rest of the population; that in Massachusetts, in 1871, "among the ignorant population, one in twenty committed crime, while among those who had a greater or less degree of education there was one felon to about a hundred and twenty-six persons;" that, out of one hundred and forty seven thousand people committed to prison by magistrates and juries in the British Islands in 1872, nearly fifty thousand were unable to read or write, and but two hundred and thirty-three had received a superior education; and that but six of the two thousand women and girls arrested in London in 1877 could do more than read and write well. That educated females really are almost entirely free from temptation to crime was proved by able English investigators more than thirty years ago, as will be seen in the *Statistical Journal.* And it may also be remarked that one of the recent volumes of the " Encyclopædia Britannica," shows that during the thirty-five years from 1841 to 1876, while the population of England and Wales increased from sixteen millions to twenty-four and a quarter, the number of criminals convicted by juries sank from twenty thousand three hundred to twelve thousand two hundred, or from one in seven hundred and eighty to one in two thousand, making about one third as much crime at the end as at the beginning of this period, during which the percentage of illiteracy was reduced to one half. In the rate of decrease of crime there are some fluctuations, owing in one instance to the demoralization caused by the potato-rot; but during the ten years from 1866 to 1876, when the system of records had become thoroughly established, there was a reduction of the number of criminals from seven to five in ten thousand, a change which may reasonably be attributed to the establishment in 1862 of a new system of education, under which the number of children in the schools doubled during these ten years. (Vol. viii., pp. 221 and 249–251.)

From these and many similar facts, I feel justified in accepting fully the results of my investigation of six hundred and twenty-three cases, and in believing education highly favorable to morality. And when we further remember how much our schools and colleges do to raise people in social station, to train them to obey laws and to respect each other's rights, and to give them such cultivated tastes as are the strongest safeguards against base indulgences, we may well say, with the Talmud, that the world is saved by the breath of school-children.

(*To be continued.*)

